# Incorporating Noise Robustness in Speech Command Recognition by Noise Augmentation of Training Data

**DOI:** 10.3390/s20082326

**Published:** 2020-04-19

**Authors:** Ayesha Pervaiz, Fawad Hussain, Huma Israr, Muhammad Ali Tahir, Fawad Riasat Raja, Naveed Khan Baloch, Farruh Ishmanov, Yousaf Bin Zikria

**Affiliations:** 1 Department of Computer Engineering, University of Engineering and Technology, Taxila 47050, Pakistan; ayesha.sip.2017@gmail.com (A.P.); fawad.hussain@uettaxila.edu.pk (F.H.); naveed.khan@uettaxila.edu.pk (N.K.B.); 2 School of Electrical Engineering and Computer Science (SEECS), National University of Sciences and Technology (NUST), Islamabad H-12, Pakistan; hisrar.dphd17seecs@seecs.edu.pk (H.I.); ali.tahir@seecs.edu.pk (M.A.T.); 3 Machine Intelligence and Pattern Analysis Laboratory, Griffith University, Nathan, QLD 4111, Australia; faraja352@gmail.com; 4 Department of Electronics and Communication Engineering, Kwangwoon University, Seoul 447-1, Korea; 5 Department of Information and Communication Engineering, Yeungnam University, Gyeongsan 38541, Korea

**Keywords:** automatic speech recognition, voice recognition, acoustic modelling, language modelling, deep learning, deep neural networks, word error rate, data science, speech command set, kaldi

## Abstract

The advent of new devices, technology, machine learning techniques, and the availability of free large speech corpora results in rapid and accurate speech recognition. In the last two decades, extensive research has been initiated by researchers and different organizations to experiment with new techniques and their applications in speech processing systems. There are several speech command based applications in the area of robotics, IoT, ubiquitous computing, and different human-computer interfaces. Various researchers have worked on enhancing the efficiency of speech command based systems and used the speech command dataset. However, none of them catered to noise in the same. Noise is one of the major challenges in any speech recognition system, as real-time noise is a very versatile and unavoidable factor that affects the performance of speech recognition systems, particularly those that have not learned the noise efficiently. We thoroughly analyse the latest trends in speech recognition and evaluate the speech command dataset on different machine learning based and deep learning based techniques. A novel technique is proposed for noise robustness by augmenting noise in training data. Our proposed technique is tested on clean and noisy data along with locally generated data and achieves much better results than existing state-of-the-art techniques, thus setting a new benchmark.

## 1. Introduction

Automatic speech recognition (ASR) is the recognition and translation of spoken language into text. An ASR system is used to estimate the most likely sequence of words for a given speech input. Over the past few decades, automatic speech recognition has been an active area of research as the technology is considered as an efficient means of human-to-human and human-to-machine communication.

Over time, the technology is getting more mature and more natural to integrate into smart devices; therefore, the use of ASR is increasing in different applications. Mobile applications like Google Assistant, Amazon’s Alexa, and Apple’s Siri [1,2] are redefining the way we interact with our smart devices. There are various reasons for this trend. First is the availability of better computational resources. Secondly, with the advancements in big data technologies, we now have access to large databases that we can use to train the generic models more efficiently. Thirdly, the number of users is growing day-by-day for smart devices such as mobile phones, smart wearables, smart homes, and infotainment systems in vehicles. To provide the best experience to the users while interacting with more advanced smart devices, it is necessary to have more robust and efficient interfaces for human-machine interaction. This will only be possible when we have standardized models for speech recognition, and such systems will ultimately facilitate all kinds of users regardless of their background, education, and lifestyle to have a natural interaction with devices.

Various applications based on speech recognition are already facilitating humankind in different tasks. Using voice search in different applications [3,4], users can search for anything using voice commands instead of using a keyboard for search. There are many examples of such applications like query search engines, driving directions, search for hotels and restaurants, and search for products on e-commerce websites.

Speech-to-Speech (S2S) translation is helpful when two people from different linguistic backgrounds need to interact with each other. They need a human interpreter who understands both languages to translate. Speech-to-speech [5,6] translation helps in automatic conversion of one language into another without the help of an interpreter, thus making communication more comfortable and natural.

Another important application of speech recognition is home automation systems. Automation is becoming an important part of our daily life, from smart homes to autonomous cars. Home automation systems [7,8] help residents remotely control various home appliances. Most of these systems have an integrated voice recognition system to operate the home appliances. Currently, voice recognition is also used in gaming applications. Instead of using a menu for the selection of items in a game, modern games use voice commands, which make such applications easier to operate.

**Figure 1:**
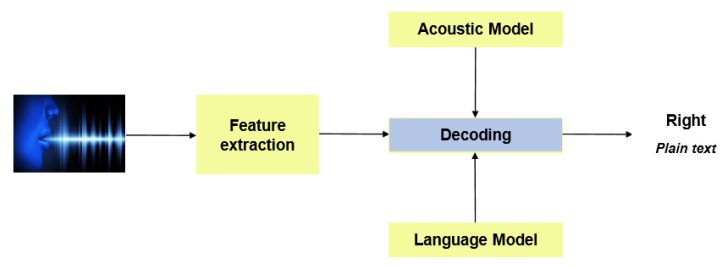
Generic model of an automatic speech recognition (ASR) system.

Acoustic feature extraction and acoustic models are two crucial parts of a speech recognition system. The acoustic features help to extract concise information from the input acoustic waveform, which is useful for recognizing the word and phonetic content of speech. Similarly, an exemplary acoustic model architecture and training method ensure that the feature vectors are robustly matched with the phoneme classes to ensure proper feature classification. The generic model of an ASR system is shown in Figure 1. The acoustic features are extracted from the raw speech signal, and the output of the acoustic model is a likely phone sequence, which corresponded to the particular speech utterance. Since the 1980s, the HMM has been a paradigm that is used to learn this mapping of phones into HMM sequences. A probabilistic model, i.e., Gaussian mixture model (GMM), maps sequences of feature vectors to a sequence of phonemes. Therefore, HMM was the state-of-the-art for a long time; however, now the deep neural networks (DNNs) have almost replaced the existing machine learning based approaches. These DDNs either learn the representations of the words after integrating in-depth features with the output of HMM-GMM based architectures or solely learn the deep features from a word-audio file or spectrogram.

Although the use of ASR in numerous applications is increasing exponentially with time, however, developing a highly efficient ASR with the least error rate is highly desired for real-time applications. Despite all the advanced algorithms that have been designed for ASR, there is still the need to improve them for real-time applications. The existing models stating state-of-the-art accuracies do not work well in a real-time environment due to the versatility of the real-time environment. The noise is one of the most significant factors that affects the accuracy of such models. To the best of our knowledge, a real-time noise factor has not been considered in the past by most of the literature, and it revolves around the learning of phones on clean data. However, a single work that embeds the noise to design its model could attain quite a bit of efficiency.

This research carries out a comprehensive study, experimentation, and comparative analysis of different machine learning and deep learning algorithms to propose the most accurate and efficient architecture for robust speech recognition in a noisy environment. In this study, various models are developed using conventional techniques such as Gaussian mixture models and hidden Markov models (GMM-HMM), deep neural networks (DNNs), conventional neural networks (CNN), and long short-term memory (LSTM). The effect of noise is highlighted by testing the proposed models on noisy data, and the impact of training on noisy data is also observed. Moreover, accent, dialect, mixed language, and non-native speech are massive problems in speech recognition; therefore, a customized dataset is collected locally in this study to test the accuracy of models in real-time applications. We also manipulate different parameters of the proposed CNN to achieve better accuracy. We evaluate the results on both versions of the speech command dataset [9,10]. We compare our results with other state-of-the-art techniques, which proves that the proposed models show significant improvement in terms of accuracy.

The rest of the paper is organized as follows. Section 2 is the literature review of various ASR systems that have already been developed. In Section 3, the design and methodology of the developed systems are discussed. It also explains all the methods used, from preprocessing to the validation of the results. In Section 4, the outcomes of different ASR systems developed during this study and various tests performed on these systems are discussed. Our research work is concluded in Section 5.

## 2. Literature Review

In order to solve the problem of ASR, various variants of DNN, CNN, and RNN have been proposed [11,12,13,14,15] for different datasets, i.e., TIMIT, Switchboard [16,17,18,19], and Aurora-4 [20,21], with the word error rate (WER) ranging from 28.9 to 4.83, in the last few years. Pete Warden contributed Speech Command Dataset Version 1 (V1) [9], an enhanced version (V2) [10] of the same dataset that was released later with additional commands.

Words for the Speech Command Dataset were chosen wisely, and most commands targeted Internet of Things (IoT) and robotics applications. Other words in the dataset were selected to cover most of the phonemes so that the models could be used as a generalized model. Pete Warden used a CNN based model for small-footprint keyword spotting [22] for evaluation of the speech command dataset and reported baseline results for both versions of the datasets. The accuracy achieved for V1 was 85.4, and for V2, it was 88.2. In the past two years, different neural network architectures trained on the same dataset were proposed; those architectures are described below.

McMahan et al. [23] used the transfer learning approach in CNN (SB-CNN [24]), ResNet [25], and DenseNet [26] for speech commands recognition. They used UrbanSound8k and a speech command dataset for their experimentation. They added multiscale input representation with dilated convolution. Moreover, they exploited different transfer learning approaches and found that pre-training on a different dataset, i.e., UrbanSound8k, produced better results than directly training on the speech command dataset. They also concluded that if transfer learning were used along with multiscale input, then only 40% of the training data were required to achieve the same results as training on 100% of the data.

Jansson [27] used CNN with one-dimensional (1D) convolutions for the recognition of speech commands by using raw waveform as the input. To increase the training data, three data augmentation techniques were used: (i) time scale shifting, (ii) amplitude scaling, and (iii) noise addition. Pseudo-labelling was also used so that the trained model could also be used to classify unlabelled data and achieve better results.

Andrade et al. [28] further improved the classification accuracy by proposing an attention based convolutional recurrent model. They used raw wav files as the input and computed spectrograms to feed the network to extract short-term and long-term dependencies. These features were then fed to the attention layer, which spotted the region of valuable information, and this information was then used as the input to the dense layer for classification.

Zhang et al. [29] explored different neural network architectures, DNN [30], CNN, CRNN [31], and LSTM [32], for the keyword spotting task for resource-constrained devices, and they also explored DS-CNN [33] for the same task and concluded that DS-CNN worked best among all in terms of accuracy within the same constraints of memory and computational power. Segal et al. [34] used a speech command dataset for pre-training of their convolutional neural network and used the YOLO (you look only once) [35] architecture by considering audio as objects. Their goal was boundary localization of utterances and then their classification.

Tang et al. [36] used ResNet for keyword spotting after successful implementation of the same on other speech domain tasks, i.e., speaker identification [37] and automatic speech recognition [38]. They took res15 as a base model to train on the speech command dataset (12 commands) along with their variants of ResNet. They magnified the residual block, explored the effect of deeper models on the word error rate (WER), and established new state-of-the-art reference models for the speech command dataset.

To the best of our knowledge, none of the above-proposed models considered training with noise-augmented audio data, for increasing noise robustness. Jansson et al. [27] used noise addition, but they did not give the result comparison with and without noise addition in the training data. Moreover, the speech commands dataset contained some noise; it was of sufficiently high SNR, and the noise level was low. This was evident from the low WER achieved on this dataset when trained on clean data using both GMM or DNN acoustic models. The following two papers explored the effect of noise by training and testing their model on noisy data. Bae et al. [39] used the capsule network, which was initially proposed for image recognition by Hinton et al. [40]. The aim of using a capsule network for speech recognition was to capture spatial relationships and pose information of features in both the time and frequency axes, which are overlooked by CNNs. By using this approach, they achieved a 11.6% and a 10.4% lesser word error rate on the clean and noisy test set, respectively, as compared to the CNN model. However, they only trained their model on the clean dataset and did not consider the noisy dataset for training.

Soni et al. [41] proposed the CNN-LSTM based neural network architecture, which consisted of two blocks. One was the convolutional enhancement block used for time-frequency masking. The other one was the LSTM based sequence block for robust speech command recognition. They trained their proposed model on the noisy dataset by adding noises provided with the speech command dataset, thus providing robustness in the model.

All of the above-described systems used CNN and its variants for their modelling of speech command recognition, whereas we used DNN and LSTM along with CNN in our proposed work.

## 3. Methodology

In this section, the details of the tools, language models, feature extraction, and acoustic models to implement different architectures are discussed.

### 3.1. Tools

Kaldi is an open-source speech recognition toolkit released under the Apache license [42]. The development of Kaldi started in 2009, and it is now one of the most famous speech recognition toolkits. The basic idea behind Kaldi was the development of an ASR toolkit that should be flexible and extensible. The main reason behind the success of Kaldi is the availability of various DNN recipes that other toolkits lack. We used the “Kaldi toolkit” for the implementation of all models except CNN. The implementation of CNN was done in MATLAB by using the “Audio Processing Toolbox.” This toolbox provides essential functions for processing audio data in bulk. The CNN models were performed on a GPU-NVIDIA TITAN Xp 12 GB, due to the training time constraint on a CPU.

### 3.2. Language Model

To define the language of the specific audio data, we needed to prepare the dictionary. The dictionary included phonemes, non-silence phonemes, and a lexicon. CMUdict initially created by the speech group at Carnegie Mellon University is typically used in Kaldi for English pronunciation, so we extracted words present in the speech command dataset from CMUdict and created a customized dictionary for our data. Table 1 defines the four files for language data.

All the unique words pronounced by the speaker were presented in the lexicon text file. Each word was associated with phonemes. There could be two or more phonemes for each word. The British and American accents have their own sets of slightly different phonemes and word pronunciations. Phonemes are the way the word is pronounced. The nonsilence_phones text file represented the unique phonemes in the lexicon text file. This was how we prepared the language data, and then by using these data, we generated a language model that was further utilized for training purposes.

### 3.3. Feature Extraction

**Figure 2:**
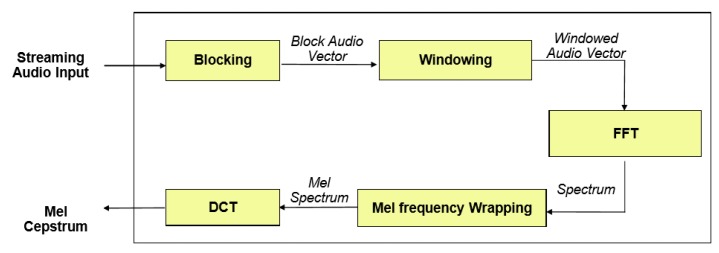
Steps involved in MFCC calculation.

Feature extraction is also known as speech parameterization, and it was used to characterize spectral features of an input audio signal in order to facilitate speech decoding. The Mel frequency cepstral coefficient (MFCC) introduced by Davis et al. [43] is one of the most popular techniques for feature extraction in speech recognition systems. The reason behind the popularity of MFCC is its ability to mimic the behaviour of the human ear. Figure 2 shows the key steps involved in the calculation of MFCC features.

#### 3.3.1. Frame Blocking

The first step involved in the process is frame blocking, in which a streaming audio signal is blocked into frames of 25 ms shifted by 10 ms.

#### 3.3.2. Windowing

After blocking, each frame is multiplied by a window using a windowing function. There are many windowing functions available in Kaldi, but usually, the Hamming window is used, as shown in Equation (1):

(1) $$w\left(n\right)=0.54-0.46\;cos\left(\frac{2\pi n}{N-1}\right)0\leq n\leq N-1$$

where Nis the number of samples in a frame, and multiplying every frame by the Hamming window reduces discontinuities at the beginning and end of each frame. This step was also required to do the frequency analysis of each frame.

#### 3.3.3. Fast Fourier Transform

Spectral analysis of speech signals showed that different timbres in a signal have different energy distributions over frequency. FFT was applied to each frame of N samples to obtain its magnitude frequency response. This process converted signals from the time to the frequency domain. FFT is a fast implementation of the discrete Fourier transform (DFT).

#### 3.3.4. Mel Frequency Warping

In this step, the magnitude frequency response resulting from FFT was multiplied by triangular bypass filters on the Mel scale to get the log energy of each bypass filter. The Mel frequency that is more discriminative at lower frequencies and less discriminative at higher frequencies mimics the non-linear perception of sound by the human ear. The human ear behaves logarithmically towards speech both on the amplitude and frequency scales, i.e., the frequency resolution is better (high) at lower frequencies and low, particularly at high frequencies. Furthermore, in the human inner ear cochlea, sound waves are transduced into electrical impulses that can be interpreted by the brain as individual frequencies of sound [44]. This is similar to how cepstral coefficients are calculated in MFCC. We could convert between Mel frequency (*m*) and frequency (*f*) in hertz by using Equations (2) and (3).

(2) $$m=2595\;{log}_{10}\left(1+\frac{f}{700}\right)$$

(3) $$f=700(10^{m/2595}-1)$$

Each filter in the triangular filter bank had a response of one at the centre frequency and decreased linearly towards zero until it reached the centre of the adjacent frequency. In Kaldi, the default number of filters is 23 because it usually gives the best results on 16 kHz speech signals.

#### 3.3.5. Discrete Cosine Transform

After log energy computations, the Mel frequency cepstrum was obtained by applying DCT on filtered results. The coefficients of the Mel frequency cepstrum are called Mel frequency cepstral coefficients (MFCC). Using DCT after FFT transforms the frequency domain into the time-like domain called the quefrency domain. In Kaldi, by default, the first 13 cepstral coefficients are kept as features.

MFCC can be directly used as features for speech recognition. However, to get better performance, various transforms are applied to the results of MFCC. One of these transformations is cepstral mean and variance normalization (CMVN) [45]. CMVN is a computationally efficient normalization technique that reduces the effects of noise. Similarly, to add dynamic information to MFCC features, first and second order deltas can be calculated. Given a feature vector *X*, first-order deltas can be calculated by using Equation (4).

(4) $$\Delta X_{t}=\frac{\Sigma_{i=1}^{n}w_{i}\left(X_{t+i}-X_{t-i}\right)}{2\Sigma_{i=1}^{n}w_{i}^{2}}$$

where *w* is the regression coefficients and *n* is the window width. Second-order deltas can be derived from first-order deltas by using Equation (5).

(5) $$\Delta X_{t}=\frac{\Sigma_{i=1}^{n}w_{i}\left({\Delta X}_{t+i}-{\Delta X}_{t-i}\right)}{2\Sigma_{i=1}^{n}w_{i}^{2}}$$

After the first- and second-order delta calculation, the combined feature vector becomes:

(6) $$\Delta X_{t}=\left[X_{t}\Delta X_{t}\Delta^{2}X_{t}\right]$$

Other feature transformation techniques used in Kaldi are linear discriminant analysis (LDA) [46], heteroscedastic linear discriminant analysis (HLDA) [47], and maximum likelihood linear transform (MLLT) [48]. These transforms can be applied individually, as well as in multiple combinations to enhance the performance of a speech recognition system. It was observed that by applying diagonalizing MLLT after LDA improved the effect of LDA (LDA + MLLT).

### 3.4. Acoustic Model

Acoustic modelling analyses the training data in terms of relevant features such as by taking different possibilities, expressing them as probabilities, and then, combining these probabilities into an HMM.

Before the training of the acoustic model, the dataset was divided into training and test sets. In this paper, four types of acoustic models, GMM-HMM, DNN-HMM, LSTM-HMM, and CNN, were trained for both versions of the dataset and their noisy versions. Moreover, the local dataset, along with standard datasets were used for the evaluation of our trained models.

#### 3.4.1. Gaussian Mixture Model Based Approaches

All the models used cepstral mean and variance normalization (CMVN) transformed MFCC features as basic features. CMVN is a well-known, computationally efficient normalization technique for robust speech recognition [49]. Distortion caused by noise contamination is minimized by CMVN, which is very helpful for robust feature extraction by linearly transforming the cepstral coefficients to have the same segmental statistics [45].

Moreover, a detailed description of the training process along with additional transformations and specifications of all variants of machine learning based models are provided below:

1. In the first step, acoustic features from training and testing data were extracted. For this study, MFCC features were used as acoustic features. The detailed process for the extraction of these features was described earlier in the feature extraction details.
2. After feature extraction, CMVN was applied to the resulting features.
3. After feature extraction and normalization, a basic monophone acoustic model called mono was trained.
4. In the next step, a basic triphone model tri1 was trained by using the same features as monophone models. The number of leaves and Gaussians used to train the network was set to 2000 and 11,000, respectively.
5. The next model (tri2a) was trained by using delta transformed features. First- and second-order Δ+ΔΔ were used as features for this model. The number of leaves and Gaussians used in training was set to 2000 and 11,000, respectively.
6. Then, the triphone model (tri2b) was trained by applying the LDA + MLLT on the acoustic features. We used the same number of leaves (2000) and Gaussians (11,000) to train the model.
7. Then, the tri2b_MMI model was trained by applying maximum mutual information (MMI) on top of LDA + MLLT with the same number of leaves and Gaussians.
8. To check the effect of boosting, boosting of 0.05 was applied on the tri2b_MMI model.
9. Then, tri2b_MPE was trained by applying maximum phone error (MPE) on top of LDA + MLLT, where the number of triphone CART leaves and Gaussians used in training was set to 2000 and 11,000, respectively.
10. The last triphone model tri3b was trained by applying LDA + MLLT + speaker adaptive training (SAT) feature transforms. Total leaves were set to 2000, and total Gaussians were set to 11,000 while training the model.
11. Similarly, MMI was applied on top of tri2b in model tri2b_MMI, and it was also applied on tri3b. tri3b_MMI used MFCC along with the LDA + MLLT + SAT + MMI feature transform.
12. Additionally, MMI including the feature space (fMMI) was applied to the tri3b_MMI model with 2000 leaves and 11,000 Gaussians while training the model.

**Figure 3:**
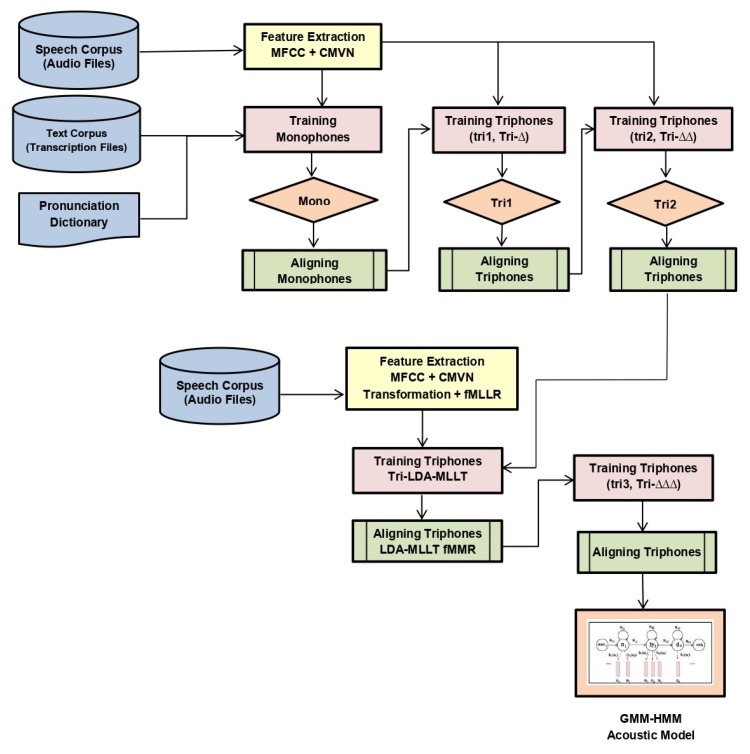
Model for the training of GMM-HMM. CMVN, cepstral mean and variance normalization; Tri, triphone; MLLT, maximum likelihood linear transform.

All the above-stated models that were trained using machine learning approaches are shown in Figure 3. We can see that it was a sequential process where alignments of each model were fed to the next model, i.e., the alignment of mono was fed in tri1, alignment of tri1 in tri2, and so on. Models were trained on features extracted from the speech corpus provided transcriptions files along with the phonetic dictionary.

#### 3.4.2. Deep Learning Based Approaches

For deep learning based approaches, we used DNN, LSTM, CNN, and their variants.

**Figure 4:**
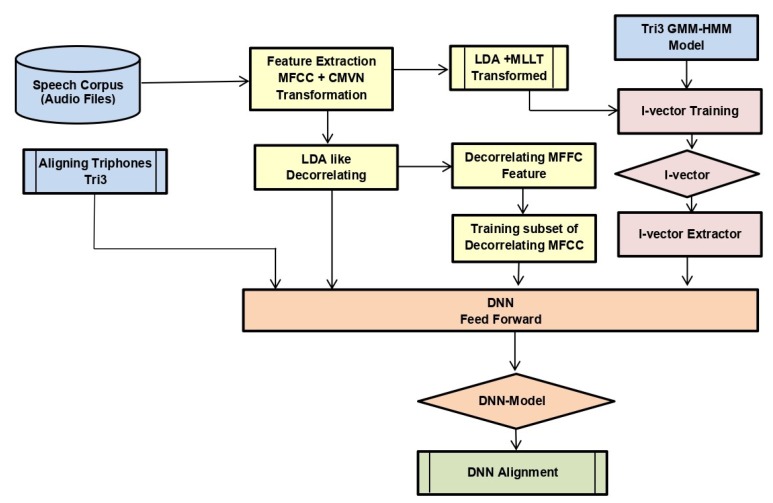
Model for the training of DNN.

**Figure 5:**
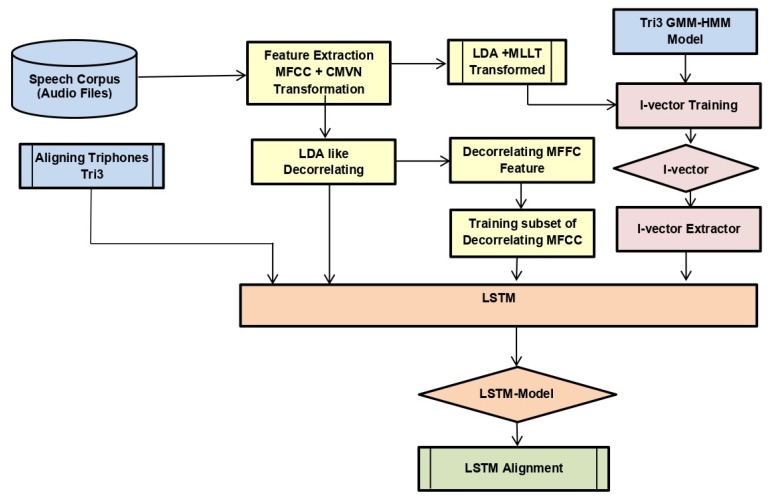
Model for the training of LSTM.

**Figure 6:**
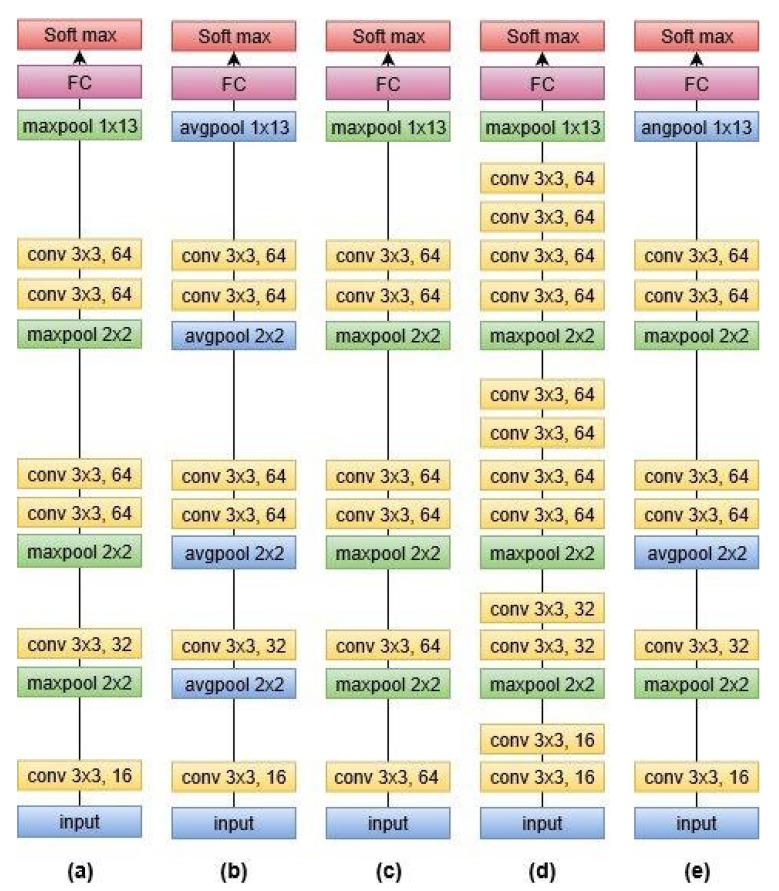
Models of CNN.

1. We used the alignments of tri3b, 40 dimension MFCC (spliced) + LDA + MLLT + fMLLR transformed features. Low-dimensional representation of the tri3 GMM-HMM model, i.e., i-vector, was also fed to DNN. The neural network saw only a window of transformed features having four frames on either side of the central frame at a time. We trained DNN with one and two layers with initial learning rate 0.01, final learning rate 0.001, input dim2000, output dim 400, number of epochs 20 with mini-batch size 128, and optimization function stochastic gradient descent. The model is shown in Figure 4.
2. We used the alignments of tri3b, 40-dimensional MFCC (spliced) + LDA + MLLT + fMLLR transformed features. Low-dimensional representation of the tri3 GMM-HMM model, i.e., i-vector, was also fed to LSTM. We trained LSTM with a three layer network with initial learning rate 0.0006, final learning rate 0.00006, input dim 1024, hidden dim 1024, RNN projection dim 256, number of epochs 10 with mini-batch size 100, and optimization function stochastic gradient descent. The model is shown in Figure 5.
3. We trained five different CNN architectures. Figure 6 shows a graphical view of these models, which are discussed below. The output was classified by using the soft-max function. Since this was a multi-class problem, soft-max helped the training to converge more quickly. The activation function at each node was the rectified linear unit (ReLU) with an initial learning rate of 0.0005. The model was trained for 25 epochs to achieve better accuracy by keeping in view the time constraint.
  (a) CNN-max
    It is a convolutional neural network with 6 convolutional layers, 4 max-pooling layers and 1 fully connected layer. All other models are the variant of this architecture.
  (b) CNN-avg
    This model was similar to (a) except max-pooling was replaced by average pooling. The purpose of this model was to report the consequence of replacing max-pooling with average pooling.
  (c) CNN-max-sameconv
    This was the same as (a), but the size of the filter was kept the same for all convolutional layers, i.e., 64. This model was used to find the outcome of varying the filter size.
  (d) CNN-max-addconv
    This was a convolutional neural network with 12 convolutional layers, 4 max-pooling layers, and 1 fully connected layer. This model would help to analyse the effect of extra convolutional layers.
  (e) CNN-max-avg
    This model was also similar to (a), and the only difference was the replacement of a few max-pooling layers with average pooling. Training such a model would depict the influence on the accuracy of the speech recognition system when both types of poolings are employed.

### 3.5. Decoding

**Figure 7:**
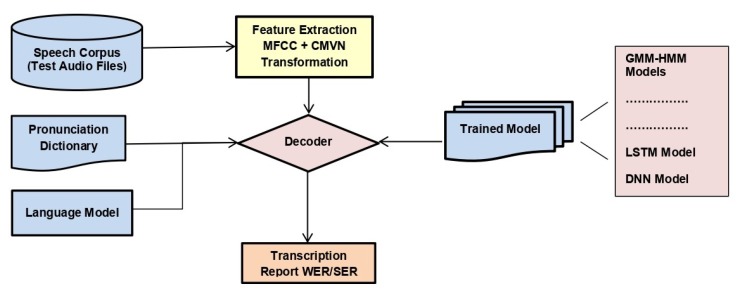
Model for decoding. WER, word error rate; SER, sentence error rate.

After training all the models, the decoder was provided with the trained model, language model, pronunciation dictionary, and features extracted from test speech corpora. The decoder matched the test sample with already learned data and produced a transcription of the audio. It also reported WER. Figure 7 illustrates the model for decoding.

## 4. Experiments

### 4.1. Datasets

For the training and evaluation of our proposed models, we used publicly available speech command datasets. For this study, we used 85% data for training, while 15% data for testing purposes.

### 4.2. Speech Command Dataset

Speech Command Dataset Version 1 (V1) contains 30 commands consisting of 64,727 utterances, uttered by 1881 speakers. An enhanced version (V2) of the same dataset was released in April 2018 with five additional commands, consisting of 105,829 utterances in total, uttered by 2618 speakers. Each audio duration was one second and comprised of a single utterance, recorded at 16kHz in wav format. We introduced six types of noises in the dataset (which were provided along with dataset); a few were real-time, while others were computer-generated, i.e., running tap, doing dishes, white noise, pink noise, etc. The duration of noisy files was more prolonged than utterances’ audio. The details of commands and noise are presented in Table 2. These datasets were acquired under a controlled environment and mainly contributed by European people.

#### 4.2.1. Local Dataset

We generated a small version of the same dataset [10] in a real-time environment based on an Asian accent for testing purposes only to check the efficiency of our trained models in a real-time and local environment. The data were acquired in a working lab environment and an outdoor environment where real-time noises were also captured along with utterances. For acquiring data from volunteers, we used an online voice recorder [50]. People from varied backgrounds contributed to our dataset. The online voice recorder by-default recorded audio at a 44kHz rate and in mp3 format. We performed pre-processing to convert these files to the same format and rate as the speech command dataset on which we trained our models so that we could test the data on already trained models. Our data consisted of a total of 2800 utterances.

#### 4.2.2. Noisy Dataset

We converted both versions of the original clean datasets into noisy datasets by adding noise to each audio file of the dataset. For this purpose, at first, we split the noisy files of different durations (provided along with dataset) into one-second audio and then randomly merged noisy files in all audio files, hence creating noisy data. We calculated the signal power level of clean audio recordings and noise recordings using the “normalize-audio” tool. The average signal power level for clean audio was −24 dB, and for noisy recordings, it was −33.3 dB. These were merged in a 1-to-1 ratio without an increase or decrease in audio volume. Therefore, the signal-to-noise ratio (SNR) of the resulting merged audio was 9.3 dB, which indicated a high noise level. Consequently, it achieved a high WER on the noisy test set in the results in Tables 3, 4, 6, and 8. We used these data in three ways, which will be explained in the next section.

### 4.3. Results and Discussion

After training different acoustic models on both versions of the dataset, extensive testing was performed on the trained models to test the efficiency of these models. As we trained different models under two categories (i) Gaussian mixture model (GMM) based techniques and (ii) deep learning based techniques, therefore, the results were tabulated in the same manner.

#### 4.3.1. Results on Gaussian Mixture Models

Ten machine learning based models already described in the above section were trained on both versions of the clean dataset and also on both versions of the noisy dataset. After successful training of all the models, each model was tested against the clean dataset, noisy dataset, and locally generated dataset. Our results showed that the performance of the tri3b model was better than the mono, tri1, and tri2 models on all three test sets. Moreover, MMI transformation on tri2b gave better performance than MPE transformation on the same. When we trained on clean data, it resulted in the clean dataset being entirely satisfactory. Still, when the same models were tested on the noisy dataset, the performance of the models was degraded as the trained models did not learn noise effectively. For more experimental insight, we also tested the same clean trained model on a locally generated test dataset that was acquired in a low noise environment. This local test set showed quite a low WER, similar to the clean test set. This was because this local recording was made in such a way that it should have minimal background noise (quite identical to the original clean test set). This test set had, however, slightly more WER as compared to the clean test set, possibly due to the non-native accent of our local speakers. Table 3 states the results of models trained on clean data, whereas Table 4 shows the results of models trained on noisy data. The maximum WER was observed with the mono model in both cases. The mono model was working only on monophones without considering the right and left context of phones. When we moved from monophones to triphones models, which took the context of right and left phones, we observed significant improvement in WER, which was further improved when we applied transformations on triphone models (the transformations applied were already mentioned in Section 3.4.1). The performance of maximum mutual information (MMI) in better than maximum phone error (MPE) and boosting factor on MMI transformation further improved the efficiency. Speaker adaptive training (SAT), along with LDA and MLLT transformations improved the WER by 14.5%.

To the best of our knowledge, we did not find any research to compare our results of machine learning based models on the same dataset. In the literature, all the researchers used deep learning techniques on the same dataset.

#### 4.3.2. Results on Deep Learning Based Models

In the same way we trained different models on GMM based techniques, we trained models on deep learning based techniques. Table 5 shows the WER when we trained our models on clean data and also tested on clean data. Our results showed a significant improvement in terms of WER when compared with other state-of-the-art models on the same dataset. The lowest WER was observed with DNN models. In the DNN model with one layer, we fed transformed features, i.e., alignments of the triphone model, as well as i-vectors of the tri3 model, rather than considering it as a black box and feeding raw input. In this way, the DNN model performed much better and showed 73% improvement in WER compared to the previously stated state-of-the-art WER [28]. CNN performed better than LSTM, but given slightly less performance than DNN. We played around with different parameters of CNN and DNN. In CNN, replacing max-pooling with average pooling reduced the accuracy, whereas the effect of outliers decreased. Replacing max-pooling with a convolutional layer also reduced the accuracy of the model, but still, it had better accuracy when it was replaced with average pooling. However, adding convolutional layers increased the accuracy, and deeper models could achieve better accuracies. We used max-pooling and average pooling alternatively on one of our models. As a consequence, we found that replacing a few max-pooling layers with average pooling layers resulted in better accuracy than replacing all max-pooling layers with average pooling layers. We also tested our clean models on noisy data, and the results are shown in Table 6. Our best model, DNN (L = 1), achieved a 13.6 % improvement in WER. Moreover, we tested our trained models on a locally acquired dataset to check the performance of our trained models in a real-time environment and to check how our trained models responded to differences in accent. The WER of models tested on locally generated data is shown in Table 7. Testing our models on a locally generated dataset resulted in 10.89% WER.

To add robustness to the noise in our models, we trained our proposed models on a noisy dataset and tested them against both clean and noisy data. Our approach of adding noise to training data was similar to the data augmentation used commonly for incorporating robustness in a model. Training data augmentation is commonly used in computer vision based applications [51]. Table 8 shows the results of noisy trained models. When we tested our noisy trained models on clean data, we achieved 5.24% WER, whereas, when tested against noisy data, the WER was 12.55. Thus, WER improved 22% compared to previous state-of-the-art models.

We can see that one or two layer LSTM deep neural networks gave optimal results. This was because of the recurrent nature of the LSTM network and the complexity of its cell structure. Furthermore, we used relatively less audio training data; therefore, one or two layers of DNN were sufficient. However, if we had to use a feed-forward deep neural network, then we may require as much as six layers, as used by [52]. Deep neural networks have been shown to give convincing improvements as compared to traditional Gaussian mixture models also on large vocabulary speech recognition tasks. The book [53] enlisted the application of DNN for large vocabulary speech recognition.

#### 4.3.3. Ablation Study

The implementation of the models was done by changing training options, the optimizer, and decoding options to check the effect of different parameters on how changing any parameter affects our WER positively. CMVN indeed reduced the effect of noise. All our input features were pre-processed using the CMVN technique. However, as we have shown in our experiments for both Gaussian mixture models and deep neural networks, that training with noisy data decreased WER on the noisy data test corpus. It clearly showed that our noise reduction technique brought additional noise robustness on top of CMVN based normalization.

Table 9 showed the WER when we changed the parameters of CNN models. CNN-max-optimized achieved the lowest WER. We optimized our CNN model by increasing the epochs to 50 and the learning rate to 0.000005. With 25 epochs, WER was 3.82, and doubling the epochs reduced WER to 3.59 for Version 1, whereas with Version 2, WER increased due to overfitting; hence, it did not result in an optimized model. For V2, we obtained the optimized model when we used previously defined training options, i.e., 25 epochs and 0.0005 learning rate with the Adam optimizer. We also observed by increasing the learning rate could slow the training process, but achieved better accuracy. Then, we applied different optimization functions to optimize our model to achieve the best performance. We used three different optimization functions, i.e., sgdm, rmsprop, and adam. The results showed that changing the optimization algorithm affected the accuracy of the model, and Adam was the best optimization algorithm. We also manipulated the effect of the beam during decoding LSTM and observed that an increasing beam reduced WER. Table 10 shows the effect on WER by changing the beam parameter.

## 5. Conclusions and Future Work

In this research, detailed experimentation and an extensive comparison of speech recognition using machine learning techniques and deep learning techniques were carried out. We trained our proposed models on both clean and noisy data and evaluated the performance of our models. Moreover, for testing the performance of our proposed models in real-time, we customized a dataset having an Asian accent with embedded noise. Our experimental evaluation on the dataset showed much better results than existing state-of-the-art techniques. After the evaluation of different models, we concluded that feed-forward DNN trained iteratively outperformed all other techniques with the minimum word error rate (WER). The performance of the DNN trained on noise augmented data, where noise was merged with the clean utterance, was found better than training the DNN on separate sets of noise and utterance files. As previously discussed, data augmentation is a well-established method for increasing the robustness of deep neural network based classifiers, especially for computer vision. Due to this, DNN learned the utterance, as well as the noise “together”, thus making it the right choice for applications in environments with background noise. Hence, this was a data augmentation technique. We concluded that it could also be effectively used for audio data applications such as speech recognition. As future work, we would like to investigate the performance of our proposed models on larger datasets with a large vocabulary. Moreover, we would like to investigate the sentence error rate (SER) of all models that are trained on isolated words, as well as phrases. For now, we augmented non-human noises in our data, but in the future, we would like to experiment with the effect of human noises in the background of real utterance.

## Figures and Tables

**Table 1:** Language data files.

File Name	Pattern of File	Pattern Example
lexicon.txt	<word> <phone 1> <phone 2>	backward B AE K W ER D
nonsilence_phones.txt	<phone>	AA, AE, AH, AO
silence_phones.txt	<phone>	SIL
optional_scilence.txt	<phone>	SIL

**Table 2:** Dataset details.

Command	Noise
core words	
down, eight, five, four, go, left, nine,	doing_the_dishes (1 min 35 s)
no, off, on, one, right, seven, six,	dude_miaowing (1 min 1 s)
stop, three, two, up, yes, zero	exercise_bike (1 min 1 s)
auxiliary words	pink_noise (1 min 0 s)
bed, bird, cat, dog, happy,	running_tap (1 min 0 s)
house, marvin, sheila, tree, wow	white_noise (1 min 1 s)
additional 5 commands in V2	
backward, follow, forward, learn, visual	

**Table 3:** WER of models trained on clean data. MMI, maximum mutual information; MPE, maximum phone error.

Model	Tested on Clean Test Set		Tested on Noisy Test Set		WER (Local Dataset)
	WER (%) (V1)	WER (%) (V2)	WER (%) (V1)	WER (%) (V2)	
mono	26.79	34.63	80.23	76.91	52.96
tri1	7.53	11.03	65.27	62.40	23.36
tri2a	7.92	11.25	65.30	63.17	22.50
tri2b	6.75	9.88	64.14	59.69	21.29
tri2_MMI	6.37	8.28	63.39	57.50	20.14
tri2b_MMI_b	6.17	8.05	63.31	57.31	20.14
tri2b_MPE	6.44	9.24	64.30	58.89	22.32
tri3b	5.77	8.72	60.41	57.72	15.14
tri3b_MMI	5.24	7.29	57.38	53.75	14.29
tri3b_fMMI + MMI	5.15	6.43	56.95	52.76	13.82

**Table 4:** WER of models trained on noisy data.

Model	Tested on Clean Test Set		Tested on Noisy Test Set	
	WER (%) (V1)	WER (%) (V2)	WER (%) (V1)	WER (%) (V2)
mono	94.20	92.61	84.47	84.40
tri1	43.32	54.90	53.65	55.32
tri2a	44.56	46.97	52.43	54.14
tri2b	47.47	45.22	42.11	54.18
tri2b_MMI	41.81	43.95	39.40	49.11
tri2b_MMI_b	40.79	43.43	39.65	49.26
tri2b_MPE	38.65	36.72	42.80	52.42
tri3b	32.56	37.87	33.40	43.11
tri3b_MMI	24.23	32.81	31.55	40.25
tri3b_fMMI + MMI	20.85	28.42	32.11	38.58

**Table 5:** WER of deep models trained and tested on clean data.

Model	WER (%) V1	WER (%) V2	Model	WER (%) V1	WER (%) V2
LSTM	4.60	6.06	Base [10]	14.6	11.8
CNN-max	3.82	3.39	Caps-inputvec4 [39]	11.6	-
CNN-avg	4.41	3.93	Caps-channel32 [39]	11.3	-
CNN-max-sameconv	4.10	3.50	Caps-outputvec4 [39]	10.5	-
CNN-max-addconv	3.69	3.15	LSTM baseline [41]	9.24	-
CNN-max-avg	3.89	3.88	Direct enhancement [41]	8.53	-
DNN (L = 1)	2.43	1.64	T-Fmasking enhancement [41]	7.1	-
DNN (L = 2)	3.00	1.78	Neural attention [28]	5.7	6.1

**Table 6:** WER of deep models trained on clean data and tested on noisy data.

Model	WER (%) V1	WER (%) V2
Caps-inputvec4 [39]	47.3	-
Caps-channel32 [39]	47.4	-
Caps-outputvec4 [39]	44.7	-
LSTM	69.09	62.79
DNN (L = 1)	38.63	25.07
DNN (L = 2)	49.13	45.09

**Table 7:** WER of models tested on locally generated data.

Model	WER (%) (Local Dataset)
LSTM	15.50
DNN (L = 1)	16.89
DNN (L = 2)	10.89

**Table 8:** WER of deep models trained on noisy data.

Model	Tested on Clean Test Set		Tested on Noisy Test Set	
	WER (%) (V1)	WER (%) (V2)	WER (%) (V1)	WER (%) (V2)
TF-Masking [41]	-	-	-	16.08
LSTM	22.09	62.04	21.08	28.26
DNN (L = 1)	5.24	55.88	14.31	12.55
DNN (L = 2)	10.91	40.29	15.91	14.44

**Table 9:** WER of models with different training options and the optimization function trained and tested on clean data.

Model	WER (%) V2	WER (%) V1
CNN-max-Optimized	12.67	3.59
CNN-max-sgdm	4.58	4.82
CNN-max-rmsprop	3.41	3.84
CNN-max-Adam	3.39	3.82

**Table 10:** WER of models with different beam parameters trained and tested on clean data.

Model	WER
LSTM (beam = 5)	6.16
LSTM (beam = 15)	6.06
LSTM (beam = 30)	5.95
